# Nasal continuous positive airway pressure improves myocardial perfusion reserve and endothelial-dependent vasodilation in patients with obstructive sleep apnea

**DOI:** 10.1186/1532-429X-12-50

**Published:** 2010-09-03

**Authors:** Patricia K Nguyen, Chandra K Katikireddy, Michael V McConnell, Clete Kushida, Phillip C Yang

**Affiliations:** 1 Department of Medicine, Division of Cardiovascular Medicine, Stanford University, Stanford, CA, USA; 2 Department of Medicine, Division of Cardiovascular Medicine, New York University, New York, NY, USA; 3 Department of Psychiatry, Stanford University Center of Excellence for Sleep Disorders, Stanford, CA, USA

## Abstract

**Background:**

Obstructive sleep apnea (OSA) has been associated with cardiovascular disease (CVD), but whether OSA is an independent risk factor for CVD is controversial. The purpose of this study is to determine if patients with OSA have subclinical cardiovascular disease that is detectable by multi-modality cardiovascular imaging and whether these abnormalities improve after nasal continuous positive airway pressure (nCPAP).

**Results:**

Of the 35 consecutive subjects with newly diagnosed moderate to severe OSA recruited from the Stanford Sleep Disorders Clinic, 20 patients were randomized to active vs. sham nCPAP. Active nCPAP was titrated to pressures that would prevent sleep disordered breathing based on inpatient polysomnography. OSA patients had baseline vascular function abnormalities including decreased myocardial perfusion reserve (MPR), brachial flow mediated dilation (FMD) and nitroglycerin-induced coronary vasodilation. Patients randomized to active nCPAP had improvement of MPR (1.5 ± 0.5 vs. 3.0 ± 1.3, p = 0.02) and brachial FMD (2.5% ± 5.7% vs. 9.0% ± 6.5%, p = 0.03) after treatment, but those randomized to sham nCPAP showed no significant improvement. There were no significant changes seen in chamber sizes, systolic and diastolic function, valvular function and coronary vasodilation to nitroglycerin.

**Conclusions:**

Patients with moderate to severe OSA had decreased MPR and brachial FMD that improved after 3 months of nCPAP. These findings suggest that relief of apnea in OSA may improve microvascular disease and endothelial dysfunction, which may prevent the development of overt cardiovascular disease. Further study in a larger patient population may be warranted.

## Background

Obstructive sleep apnea (OSA) has been associated with an increased incidence of cardiovascular disease (CVD) and these patients are, thus, likely to have a high burden of subclinical disease [1,2]. However, the extent of subclinical CVD has not been systematically evaluated. Previous studies have used single measures of subclinical disease [3-7]. In addition, whether OSA plays an independent role in the development of CVD remains controversial since *most *previous studies are cross sectional and not randomized, and, thus, may not adequately control for confounding factors.

We use multi-modality cardiovascular imaging (CVI) to evaluate subclinical CVD in patients with OSA before and after randomization to active or sham nasal continuous positive airway pressure (nCPAP). We will (1) determine the frequency of subclinical CVD using multi-modality imaging in adults with newly diagnosed moderate to severe OSA and (2) test the hypothesis that nCPAP therapy, the standard treatment for OSA, improves these abnormalities.

## Methods

### Subjects

A total of 35 consecutive patients were recruited from the patient population seen at the Stanford Sleep Disorders Clinic. The inclusion criteria include: 1) newly diagnosed moderate to severe OSA as defined by the American Academy of Sleep Medicine [8], 2) RDI ≥ 15 events per hour by *inpatient *polysomnography, and 3) Epworth Sleepiness Scale score > 10. The exclusion criteria include: 1) prior treatment for OSA, 2) an oxygen saturation < 75% for > 10% of the diagnostic sleep study or < 75% for > 25% of the first 4 hours of the diagnostic sleep study, 3) clinical symptoms or diagnosis of coronary artery disease, congestive heart failure, cardiac rhythm disturbance, Raynaud's disease (which is a contraindication for flow mediated dilation), respiratory disease, diabetes, chronic neurological disorders, cancer not in remission, and renal failure, 4) metal objects, devices or implants in or on the body including pacemakers, aneurysm clips, prostheses, bullets, buckshot, shrapnel, and any metal fragments from working around metal (which are contraindications for cardiovascular magnetic resonance imaging) and 5) contraindications to adenosine or nitroglycerin. Seven participants failed the initial screening for eligibility, and 8 withdrew prior to randomization. The Stanford Institutional Review Board approved the study. All subjects gave written informed consent.

### Recruitment, Randomization and Blinding

Subjects were identified for recruitment after undergoing a standard overnight *inpatient *respiratory polysomnographic sleep study. The diagnostic polysomnogram served as the baseline measure. Patients also completed a standard questionnaire to evaluate the degree of sleepiness and underwent baseline echocardiography, cardiac magnetic resonance imaging, and vascular ultrasound. After baseline assessments, subjects were randomized to either active (therapeutic) or sham (sub therapeutic) nCPAP and then admitted for a second night of polysomnography for nCPAP titration. During the nCPAP titration night, if patients were randomized to active nCPAP, pressures were varied throughout the night to control the patients' sleep disordered breathing. For those on sham nCPAP, pressures fluctuated slightly but no more than 0.5 cm H_2_O pressure, which was achieved by inserting a flow restricting connector at the machine outlet and six extra 4 mm holes in the collar of the main tubing at the end of the mask to allow air to escape and to prevent re-breathing of carbon monoxide. A certified technician reviewed the polysomnograms the next day to determine the optimal therapeutic pressure to control snoring and sleep apnea for patients in the active nCPAP group. Patients assigned to sham nCPAP received a similar nCPAP device but the machine delivered air pressure insufficient to prevent sleep disordered breathing. In all other ways, the nCPAP machines were similar. The patients, technicians conducting the titration studies and investigators assessing the imaging studies were not aware of treatment group assignments. Thus, the study was effectively double-blind.

Within 7 days after the assessment, the patients were given their nCPAP machines and instructed how to use the machines at home. Study staff contacted patients monthly to assess adherence to the devices. Patients returned after three months for repeat polysomnogram and multi-modality CVI studies. Information on compliance was downloaded from the assigned nCPAP machines at the end of the study.

### Polysomnography

Overnight respiratory polysomnographic sleep studies were performed at baseline, for nCPAP titration, and after treatment. An obstructed breathing event consisted of an obstructive apnea, hypopnea, or respiratory effort-related arousals [9]. Moderate or severe apnea was defined as a respiratory disturbance index > 15 events per hour of sleep.

### Echocardiography

Echocardiographic images were obtained in standard views by the same experienced sonographer (Hewlett Packard, Sonos, 5500). Left ventricular diastolic function and pulmonary arterial pressure was assessed by Doppler echocardiography in accordance with the American Society of Echocardiography recommendations [10].

### Cardiovascular magnetic resonance

Cardiovascular magnetic resonance (CMR) included an assessment of structure and function, adenosine stress myocardial perfusion, and coronary artery vasodilation to nitroglycerin (NTG). Imaging analysis was performed using Report Card, the GE software.

Scans were performed using a 1.5T Signa MR scanner (GE Healthcare, Milwaukee, WI) equipped with high-performance gradients (40 mT/m, 150 mT/m/ms). A commercial 4-channel cardiac phased-array surface coil provided signal reception (GE Healthcare, Milwaukee, WI). A real-time interactive system (iDrive, GE Healthcare, Milwaukee, WI) was used for localization. Assessment of cardiac function was obtained using an ECG-triggered retrospectively gated cine SSFP sequence (20 phases per cardiac cycle, TR = 3.6 ms, TE = 1.6 ms, FOV = 280 to 390 mm and flip angle = 40°). First-pass myocardial perfusion imaging was performed using a segmented echo-planar imaging pulse sequence with a notched saturation pulse [11]. For stress imaging, adenosine was administered intravenously at a rate of 140 mcg/kg/min for 4 minutes, followed by first-pass myocardial perfusion imaging during intravenous injection of 0.1 mmol/kg gadolinium-DPTA at a rate of 5 ml/s. Rest perfusion images were acquired approximately 10 minutes after stress images. The following perfusion pulse sequence parameters were used: TR = 2.4 ms, TE = 1.2 ms, inversion time = 158 to 211 ms, echo train length = 4 to 8 ms, FOV = 34 to 37 × 25 to 27 cm, matrix = 128×128, flip angle = 25°, and slice thickness = 10 mm. The rest perfusion images were generally acquired with the same graphic prescription used for the stress images. For analysis of myocardial perfusion, signal intensity was determined for each of the three contiguous slices representing the base, mid and apex of the left ventricle. The signal intensity before contrast agent administration was subtracted, and the upslope of the resulting signal intensity time curve was determined. A perfusion score was generated by adding the upslope of each of the three slices. The myocardial perfusion reserve (MPR) was calculated as the ratio between the myocardial perfusion score at stress divided by the myocardial perfusion score at rest.

NTG-induced coronary vasodilation was then performed. Using a real-time interactive system, in-plane views of the right coronary artery (RCA) were prescribed. A cross-sectional view of the most linear portion of the proximal to mid RCA was prescribed. Multi-slice high-resolution spiral coronary magnetic resonance angiography was performed with cardiac gating, breath-holding, and acquisition during diastole (FOV = 22 cm, in-plane spatial resolution = 0.7 mm, slice thickness = 5 mm, 3 slices, TR = 1 heart beat, TE = 2.5 ms, 18 interleaves, and flip angle = 60°). Cross-sectional spiral high-resolution coronary magnetic resonance angiography images [12] were acquired before and 5 minutes after administration of 0.4 mg sublingual NTG while the patient was inside the magnet.

Using the cross-sectional RCA images, the most circular and corresponding slices were identified on the pre- and post-NTG images. These images were all pooled and then randomized, with neither patient nor NTG information provided on the images. We used a custom designed software program to analyze the cross sectional images. Images were magnified two-fold, and an ovoid region of interest tool was used to trace around the RCA, yielding the cross-sectional area.

### Vascular Ultrasound

Endothelial function was assessed non-invasively using vascular ultrasound to measure brachial artery flow mediated dilation (FMD) following reactive hyperemia in accordance with published guidelines [13]. Studies were performed by one of two trained operators. Studies were performed at the same time of day and in the fasting state. Vasoactive medications were held 24 hours prior to the study.

Brachial artery diameters were measured using an automated software system to detect near and far wall edges and measure vessel diameter for each frame in the 10-second loop (Vascular Analysis Tools, Medical Imaging Applications, Iowa, USA). All analyses were performed by one of two trained operators. Previous reproducibility studies indicate high operator agreement.

## Sample Size Calculation

The sample size was estimated based on a previous randomized study comparing FMD in patients with moderate to severe OSA randomized to 3 months of nCPAP and no nCPAP [14]. FMD was significantly lower in patients randomized to nCPAP (nCPAP 8.9 ± 1.9%, no nCPAP 5.0 ± 0.7%, p = 0.02). Using a two tailed α = 0.01 and power of 0.90, an attrition rate of 20%, the estimated sample size is 10 subjects per each group (total number of subjects is 20).

## Statistical Analysis

Continuous variables were reported as means with standard deviations. Categorical variables were reported as frequencies and counts. Standard thresholds for abnormal values were used for all parameters. An MPR < 2.5 was considered abnormal. This cut off value is chosen based on a review of studies by Koch, which showed that arteries with significant narrowing (> 70%) had a flow reserve less than 2.5 [15]. An MPR of < 2.5 was, therefore, selected for the most significant flow impairment. A global MPR < 2.5 was also used as the cutoff in the Multi-Ethnic Study of Atherosclerosis (MESA), a population-based prospective cohort study of subclinical cardiovascular disease and its progression [16]. A change of < 9% and < 15% in the brachial artery diameter after FMD and in the coronary artery cross sectional area after NTG were considered abnormal, respectively. These cutoff values were chosen based on a study in 228 non diabetic subjects who were matched for age and gender. The study showed that flow mediated dilation (8.5% ± 5.3% versus 11.7% ± 6.3%, p < 0.001) and NTG-mediated vasodilation (14.9% ± 6.0% vs. 18.5% ± 7.8%, p = 0.003) were both impaired in hypertensive compared to normotensive individuals [17].

The differences between clinical characteristics and compliance between patients receiving active and sham nCPAP were assessed by unpaired Student's t test and Chi square test for continuous and categorical variables, respectively. Multi-modality imaging parameters were compared before and after treatment using paired Student's t test and McNemar's test for continuous and categorical variables, respectively. For the analysis of diastolic function parameters using the McNemar's test, categories were collapsed into normal and abnormal. Specifically, the following were considered normal: E/A > 0.75 and < 1.5, DT = 140 - 240 ms, E prime < 8 cm/s and a pulmonary vein pattern that was not diastolic predominant. Other values were considered abnormal. Linear regression analysis was performed to determine the relationship between the RDI and the following variables measured at baseline: 1) MPR, 2) NTG-induced coronary vasodilation, and 3) FMD. A two-tailed p value < 0.05 was considered statistically significant. Statistical analyses were performed with SPSS software version 13.0 (SPSS, Inc. Chicago, IL).

## Results

### Demographic and Clinical Characteristics

There were no significant differences between baseline characteristics between the two groups (Table 1). The average age of subjects was 53.4 ± 11.2 years. The majority of patients were Caucasian males who were overweight and nonsmokers with hypertension. There were no significant differences between patients randomized to active vs. sham nCPAP in the RDI (37.7 ± 19.4 vs. 36.2 ± 17.0, p = 0.87) and apnea hypopnea index (38.8 ± 21.38 vs. 31.6 ± 11.14, p = 0.79). There was no significant difference after adjustment for sleep duration in patients randomized to active vs. sham nCPAP for obstructive apneas (84.4 ± 89.0 vs. 35.3 ± 29.7, p = 0.12), total apneas (85.1 ± 89.5 vs. 39.8 ± 28.8, p = 0.17) and total hypopneas (149.2 ± 33.8 vs. 130.0 ± 63.2, p = 0.41), respectively. Except for changes in high density lipoprotein levels, there were no significant differences between the two groups before and after nCPAP (Table 2).

**Table 1:** Demographics and Clinical Characteristics

	Sub therapeutic nCPAP (n = 10)	Therapeutic nCPAP (n = 10)	All Patients (n = 20)	p value
Age, years	53.9 ± 10.8	52.9 ± 11.6	53.4 ± 11.2	0.85
Male	10/10	8/10	18/20	0.47
Caucasian	6/10	6/10	12/20	1.00
BMI, kg/m^2^	29.6 ± 5.6	30.1 ± 4.7	29.8 ± 5.2	0.85
Smoking	0/10	1/10	1/20	1.00
HTN	10/10	10/10	20/20	1.00
DM	1/10	0/10	1/20	1.00
Dyslipidemia	5/10	4/10	9/20	1.00
# Awakenings During Night	1.35 ± 0.98	2.1 ± 0.6	1.7 ± 0.9	0.07
# Hours of Sleep on Weekday Nights	7.15 ± 1.32	7.53 ± 0.81	7.34 ± 1.11	0.48
# Hours of Sleep on Weekend Nights	6.93 ± 2.43	7.7 ± 1.12	7.34 ± 1.11	0.40
# Naps ≥ 5 minutes Per Week*	1.7 ± 2.25	1.1 ± 1.46	1..4 ± 1.92	0.51
Feels Unrested During Day*	1.8 ± 1.2	1.2 ± 0.87	1.15 ± 1.2	0.25
Feel Excessively Sleepy During Day*	1.8 ± 0.87	1.3 ± 0.64	1.56 ± 0.80	0.18
Do Not Get Enough Sleep*	2.4 ± 0.8	2.0 ± 0.89	2.2 ± 0.87	0.33
Feel Excessively Fatigued During Day*	2.3 ± 1.27	1.6 ± 0.92	1.95 ± 1.16	0.20
# Caffeinated Beverages Per Week	1.9 ± 1.5	1.05 ± 0.57	1.5 ± 1.2	0.13
# Alcoholic Beverages Per Week	4.3 ± 5.2	2.1 ± 5.01	3.2 ± 5.2	0.37

p values reflect differences in scores between Post nCPAP minus Baseline for the Active vs. Sham Groups

* 0 = never; 1 = rare (1/month or less); 2 = sometimes (2-4/month); 3 = often (5 - 15/month); 4 = almost always (16-30/month)

BMI: body mass index, HTN: hypertension, DM: diabetes

**Table 2:** Clinical Parameters Before and After Treatment

	Baseline			Treatment			Before vs. After Treatment Pairwise Comparison (p value)	
	Active	Sham	p	Active	Sham	p	Active	Sham
Average SBP, mmHg	121.0 ± 11.6 (10/10)	127 ± 10.3 (10/10)	0.26	126.0 ± 15.8 (10/10)	130.4 ± 14.2 (10/10)	0.54	0.55	0.33
Average DBP, mmHg	75.2 ± 11.1 (10/10)	78.9 ± 11.4 (10/10)	0.11	83.9 ± 10.6 (10/10)	78.7 ± 11.2 (10/10)	0.97	0.34	0.06
Fasting glucose	99.5 ± 17.1 (10/10)	91.1 ± 13.9 (9/10)	0.27	101.6 ± 18.7 (10/10)	91.6 ± 19.3 (9/10)	0.28	0.96	0.92
2 hour Fasting Glucose	142.3 ± 64.3 (10/10)	109.1 ± 58.6 (9/10)	0.26	140.5 ± 38.4 (10/10)	120.0 ± 55.6 (9/10)	0.39	0.89	0.58
LDL, mg/dl	105.3 ± 16.7 (9/10)	109.4 ± 30.4 (8/10)	0.75	114.6 ± 34.3 (9/10)	107.1 ± 40.0 (8/10)	0.89	0.45	0.75
HDL	47.1 ± 18.5 (10/10)	42.2 ± 15.0 (10/10)	0.56	42.0 ± 15.9 (10/10)	40.1 ± 9.0 (10/10)	0.78	0.04	0.86
Cholesterol/HDL	4.1 ± 1.3 (10/10)	5.0 ± 2.4 (10/10)	0.31	4.9 ± 1.8 (10/10)	4.6 ± 1.7 (10/10)	0.86	0.12	0.15
Triglycerides	148.0 ± 128.4 (10/10)	172.9 ± 200.4 (10/10)	0.76	145.8 ± 86.0 (10/10)	173.9 ± 206.8 (10/10)	0.71	0.96	0.92

SBP: systolic blood pressure, DBP: diastolic blood pressure, LDL: low density lipoprotein, HDL: high density lipoprotein

### Polysomnography

Compliance with nCPAP was equivalent in both groups with the average use per night of 5.1 ± 1.9 hours and 4.9 ± 2.3 hours in the active and sham groups, respectively (p = 0.9). Changes in polysomnography measurements before and after treatment are shown in Table 3.

**Table 3:** Polysomnography Before and After Treatment

	Baseline			Treatment			Before and After Treatment Pairwise Comparison (p value)	
	Active	Sham	p	Active	Sham	p	Active	Sham
RDI	37.7 ± 19.4	36.2 ± 17.0	0.87	2.2 ± 1.5	37.4 ± 23.3	0.0003	0.0004	0.91
Obstructive events	84.4 ± 89	35.3 ± 29.7	0.13	0.8 ± 1.6	71.2 ± 115	0.08	0.02	0.4
OEI	13.6 ± 15.2	7.4 ± 5.9	0.21	0.11 ± 0.22	10.6 ± 16.4	0.07	0.03	0.59
AHI	38.8 ± 21.38	31.6 ± 11.14	0.79	2.2 ± 1.5	37.4 ± 23.3	0.0003	0.0004	0.89
Sleep Efficiency	80.2 ± 8.6	73.8 ± 13.1	0.23	87.4 ± 6.8	86.7 ± 7.6	0.84	0.02	0.002
Sleep Latency (min)	17.2 ± 19.6	16.7 ± 17.0	0.77	8.3 ± 9.5	9.54 ± 6.6	0.89	0.1	0.03
Total Sleep Time, min	379.2 ± 48.8	291 ± 81.5	0.01	408.2 ± 51.3	377.9 ± 66.7	0.27	0.18	0.04
NREM I, % of TST	10.84 ± 8.0	12.5 ± 10.9	0.65	9.5 ± 5.0	3.2 ± 0.6	0.6	0.50	0.20
NREM II, % TST	67.0 ± 11.3	58.2 ± 27.4	0.35	69.6 ± 6.3	70.8 ± 4.5	0.62	0.60	0.80
NREM III, % TST	2.7 ± 5.8	0.94 ± 1.9	0.39	1.6 ± 1.9	2.0 ± 0.8	0.74	0.62	0.16
NREM, IV, % TST	0.05 ± 0.15	0.71 ± 1.4	0.17	0.9 ± 1.7	0.2 ± 0.1	0.14	0.17	0.37
REM, % of TST	18.9 ± 7.4	14.1 ± 7.3	0.18	17.7 ± 5.1	2.8 ± 0.4	0.40	0.70	0.38
Minimal nocturnal SaO_2_	82.3 ± 5.5	83.8 ± 11.5	0.17	93.4 ± 2.3	86.1 ± 7.8	0.01	0.13	0.28
SaO2 < 90%, % of TST	8.0 ± 14.4	4.1 ± 0.3	0.28	0.07 ± 0.2	5.7 ± 7.4	0.03	0.0003	0.05

RDI: respiratory disturbance index defined as the number of events that distrupt sleep divided by the sleep duration in hours;

OEI: obstructive events index defined as the number of obstructive events divided by the sleep duration in hours.

AHI: apnea-hypopnea index defined as the number of apnea-hypopnea events divided by the sleep duration in hours.

### Echocardiography

Echocardiography assessment was performed successfully in 20/20 patients, respectively. Seven patients with OSA had at least one diastolic parameter that was suggestive of diastolic dysfunction. Four patients had significant valvular abnormalities including 3 patients with significant aortic regurgitation and one patient with significant mitral regurgitation. Most patients had inadequate tricuspid regurgitation to estimate right ventricular systolic pressures, but of those with measurable values, all (8/8) had normal pulmonary pressures. Patients randomized to active nCPAP had significant improvement in the E/A ratio after treatment; whereas, those randomized to sham nCPAP did not. Changes in echocardiographic parameters before and after nCPAP are shown in Table 4.

**Table 4:** Diastolic Function and Pulmonary Arterial Pressure by Echocardiography Before and After Treatment

	Baseline		Treatment		Before vs. After Treatment: Pair-wise Comparison (p value)	
	**Active**	**Sham**	**Active**	**Sham**	**Active**	**Sham**
						
E wave, cm/s						
≤ 1 cm/s	90% (9/10)	100%(10/10)	90% (9/10)	80% (8/10)		
> 1 cm/s	10% (1/10)	0% (0/10)	10% (1/10)	20% (2/10)		
p value	1.0		1.0		1.0	0.25
E/A ratio						
≤ 0.75	40% (4/10)	10% (1/10)	0% (0/10)	20%(2/10)		
> 0.75 & < 1.5	30% (3/10)	90%(9/10)	90%(9/10)	60%(6/10)		
≥1.5	30%(3/10)	0%(0/0)	10% (1/10)	20%(2/10)		
p value	1.0		1.0		0.03	0.25
DT						
< 140 ms	0 (0/10)	0 (0/10)	0 (0/10)	0 (0/10)		
140-240 ms	60% (6/10)	80% (8/10)	90% (9/10)	70%(7/10)		
> 240 ms	40% (4/10)	20% (2/10)	10%(1/10)	30%(3/10)		
p value	0.62		0.33		1.00	1.00
E'						
≤ 8 cm/s	11% (1/9)	20%(2/10)	11% (1/9)	40% (4/10)		
> 8 cm/s	89% (8/9)	80%(8/10)	89%(8/9)	60%(6/10)		
p value	0.92		0.30		1.00	0.25
E/e'						
< 8	66.7% (6/9)	50% (5/10)	44% (4/9)	60% (6/10)		
8-15	33.3%(3/9)	50%(5/10)	56%(5/9)	40%(4/10)		
> 15	0% (0/10)	0% (0/10)	0% (0/10)	0% (0/10)		
p value	0.90		0.66		0.5	0.5
S/D ratio						
S > D	50%(4/8)	40% (2/5)	5/8 (50%)	40% (2/5)		
S = D	50%(4/8)	60% (3/5)	3/8(30%)	60% (3/5)		
S < D	0% (0/8)	0 (0/5)	0 (0/8)	0 (0/5)		
p value	1.00		0.59		1.00	1.00

E: peak velocity associated with early mitral filling, A: peak velocity associated with the atrial kick, DT: deceleration time, E': E prime by tissue doppler, S: peak systolic velocity associated with the pulmonary vein flow, D: peak diastolic veolicty associated with pulmonary vein flow

### Cardiovascular Magnetic Resonance

Assessment of cardiac structure and function was successfully performed in 20/20 patients with no significant differences in cardiac structure and function between the two groups (Table 5). Except for LV ejection fraction, there was no significant change in chamber sizes, end diastolic volumes, right ventricular ejection fraction, and LV mass after 3 months of therapy.

**Table 5:** Cardiac Structure and Function, Myocardial Perfusion Reserve and Coronary Artery Vasoreactivity by Magnetic Resonance Imaging

	Baseline			Treatment			Before vs. After Treatment Pairwise Comparison (p value)	
	Active	Sham	p	Active	Sham	p	Active	Sham
LV Mass	106.1 ± 28.9	108.3 ± 23.6	0.86	111.34 ± 39.8	118.4 ± 25.0	0.66	0.36	0.18
LV Mass/BSA	52.2 ± 11.4	49.3 ± 7.9	0.54	56.6 ± 18.4	69.9 ± 32.2	0.98	0.38	0.15
RVEDV	161.1 ± 28.6	171.8 ± 35.4	0.49	165.8 ± 38.1	181.6 ± 54.2	0.48	0.40	0.40
RVEDV/BSA	79.7 ± 13.0	79.1 ± 15.8	0.93	81.7 ± 79.4	82.6 ± 22.5	0.92	0.45	0.53
LVEDV	148.3 ± 32	158.3 ± 31.1	0.51	151.1 ± 30.5	163.4 ± 38.7	0.46	0.63	0.39
LVEDV/BSA	73.0 ± 13.5	72.3 ± 9.6	0.88	74.7 ± 13.4	74.4 ± 14.2	0.96	0.47	0.57
RV EF	58.9 ± 6.9	56.7 ± 9.0	0.57	55.5 ± 5.7	54.0 ± 11.4	0.80	0.26	0.43
LV EF	68.9 ± 7.1	67.4 ± 8.2	0.69	71.5 ± 4.6	65.1 ± 7.5	0.04	0.34	0.31
MPR	1.5 ± 0.5	2.0 ± 1.2	0.38	3.0 ± 1.3	2.5 ± 1.1	0.49	0.02	0.38
Change in CSA RCA, %	14+8	20 ± 10	0.16	18 ± 15	15 ± 8	0.58	0.46	0.23

LV: left ventricle, BSA: body surface area, RV: right ventricle, EDV: end diastolic volume, EF: ejection fraction, MPR: myocardial perfusion reserve, CSA: cross sectional area, RCA: right coronary artery

Adenosine stress perfusion CMR was successfully performed in 16/20 patients. No patients had adverse cardiovascular events during adenosine stress study. No regional myocardial perfusion defects were noted by qualitative visual assessment in either group.

Baseline MPR was abnormal in patients with sleep apnea with no significant differences between subjects randomized to active and sham. There was no significant correlation between baseline RDI and MPR (r = 0.32, p = 0.24). Three months after randomization, the MPR increased significantly in patients randomized to active nCPAP (1.5 ± 0.5 vs. 3.0 ± 1.3, p = 0.02); however, it did not change significantly in patients randomized to sham nCPAP (2.0 ± 1.2 vs. 2.5 ± 1.1, p = 0.38 (Table 5).

### NTG Coronary Vasodilation

At baseline, the mean percent vasodilation to NTG was abnormal, but did not significantly differ between the two groups (Table 5). There was no significant correlation between baseline RDI and NTG coronary vasodilation (r = -0.14, p = 0.64). There was no significant difference in coronary vasodilation after 3 months of therapy in either the active or sham groups.

### Vascular Ultrasound

Assessment of brachial artery FMD by vascular ultrasound was successfully performed in 18/20 patients. Patients with OSA had impaired FMD with no significant differences in brachial artery FMD at baseline in the active vs. sham groups (Table 6). There was no significant correlation between baseline RDI and FMD (r = 0.08, p = 0.78). Three months after randomization, FMD increased significantly and even normalized in the active group (2.5 ± 5.7 vs. 9.0 ± 6.5, p = 0.03); however, there was no significant improvement in the sham group (4.0 ± 2.4 vs. 2.7 ± 2.4, p = 0.47). No significant difference was detected in either group in NTG induced vasodilatation of the brachial artery following 3 months of therapy.

**Table 6:** Brachial Artery Reactivity by Vascular Ultrasound at Baseline and On Treatment

	Baseline			Treatment			Before vs. After Treatment Pairwise Comparison (p value)	
	Active	Sham	p	Active	Sham	p	Active	Sham
% Brachial FMD	2.5 ± 5.7	4.0 ± 2.4	0.53	9.0 ± 6.5	2.7 ± 2.4	0.01	0.03	0.24
% NTG induced vasodilation	7.0 ± 5.0	9.0 ± 5.0	0.38	9.0 ± 7.0	7.0 ± 5.0	0.47	0.47	0.38

FMD: flow mediated dilation; NTG: nitroglycerin

## Discussion

This is the first study to provide a comprehensive evaluation of subclinical CVD using multi-modality CVI in patients with OSA. As expected in asymptomatic patients with moderate to severe OSA and no known cardiovascular disease, most patients did not have significant abnormalities of cardiac structure and contractility. Patients with OSA, however, had abnormal MPR and brachial FMD, suggesting these patients have microvascular disease and endothelial dysfunction, respectively. These abnormalities improved after 3 months of active nCPAP, providing further evidence that OSA may contribute to the development of CVD.

### Preserved Cardiac Structure and Function

Previous studies have shown that OSA adversely affects the structure and systolic function of the left [18-20] and right ventricle [19,21]. Patients with OSA have recurrent increases in left ventricular (LV) afterload during sleep that results from large negative intra-thoracic pressure swings, hypoxemia and arousals from sleep. Recurrent LV strain over several hours of apneas may result in chronic LV dysfunction. Increased sympathetic activity and hypoxemia also contribute to LV dysfunction. Improvement has been reported after nCPAP and may be related to a reduction in nocturnal afterload. LV dysfunction, however, appears to be a late complication and was observed in < 10% of cases in a previous study [18] and was not observed in this study.

Other abnormalities have been reported in patients with OSA including an increase in LV mass [22,23] and diastolic dysfunction [23]. However, a recent report [23] has shown that these abnormalities did not correlate with the apnea hypopnea index (AHI) or with oxygen saturation but were associated with age, hypertension and obesity. Consistent with these findings, most patients in our study whose average age was 55 years old and who were not obese (average body mass index < 30) had normal LV mass and diastolic function. Although all patients had a history of hypertension, the average blood pressure was normal and the majority of patients had good control of their blood pressure with prescribed medications.

Similarly, RV dysfunction may be a result of chronic intermittent hypoxia and hypercapnia during apneic episodes. RV dysfunction may also be secondary to left ventricular dysfunction as a result of increased afterload and sympathetic activity, which causes secondary hypertension. RV dysfunction [21] and elevated RVSP [24], however, is an uncommon finding in patients with sleep apnea and was not found in our study.

Estimates of ejection fraction, however, may not be sensitive enough to detect early changes related to obstructive sleep apnea. Previous studies [19] have shown impairment of the left and right ventricular myocardial performance index, which is derived by measurement of the isovolumic contraction and relaxation times and valvular ejection times. Improvement correlated well with AHI, independent of confounders. Reduced systolic and diastolic velocities [25] of the left and right ventricle have also been observed in patients with OSA and were noted to improve after six months of CPAP. Future studies are needed to determine the clinical utility of these measures in the assessment of patients with sleep apnea.

Microvascular Disease and Endothelial Dysfunction in Patients with OSA

Microvascular disease and endothelial dysfunction are the earliest manifestations of coronary heart disease and can be found in patients without obstructive coronary artery disease (CAD) or myocardial disease. Coronary microvascular dysfunction has been previously described in patients with risk factors for CAD. In a previous study, coronary flow reserve was reduced by 21% in smokers and normalized with vitamin C administration, an anti-oxidant [26]. Impairment in coronary flow reserve has also been shown in asymptomatic subjects with hypercholesterolemia [27] and angiographically normal coronary arteries with demonstrated reversibility with cholesterol lowering strategies [28]. Similarly, in our study, patients with OSA with no documented obstructive CAD and no myocardial disease had impaired MPR. MPR improved in patients randomized to active but not sham nCPAP, suggesting that the presence of microvascular disease is due to OSA and not to the presence of cardiovascular risk factors in these patients. To our knowledge, this is the first study to show that patients with OSA have microvascular disease measurable by CMR MPR, which improves with therapeutic nCPAP.

Similarly, endothelial dysfunction has been described in patient with coronary risk factors as well as those with OSA. Previous studies have shown that patients with OSA have impaired brachial artery dilation to acetylcholine, an endothelial-dependent stimulus, compared with control subjects [29,30]. A recent study showed that endothelial function in OSA patients improved after 3 months of nCPAP [31]. This study also demonstrated that resting nitric oxide (NO) production was higher after CPAP. Other studies have confirmed improvement in peripheral endothelial function as early as two weeks after CPAP and increases in plasma levels of NO have been observed as early as one night after nCPAP [32,33]. Our study confirms these findings. In this study, patients with OSA had decreased brachial FMD. Patients randomized to active nCPAP showed improvement in brachial FMD; whereas, those randomized to sham nCPAP showed no significant change. At baseline and at 3 months, there were no significant changes in cardiac risk factors except for high density lipoprotein, which decreased in both groups. The significance of which is unclear but may be related to inactivity secondary to nCPAP usage. This study supports that relief of apneic events by nCPAP in patients with sleep apnea improves endothelial function.

Several mechanisms [34] have been suggested to explain the relationship between OSA and vascular dysfunction. Evidence suggests that recurrent apnea-hypopnea events in OSA patients are associated with repetitive hypoxemia and reoxygenation, resulting in increased production of reactive oxygen species. This leads to increased intra and extra cellular oxidative stress and breakdown of NO, resulting in vascular dysfunction. The relationship between apnea and vascular dysfunction was further supported in a recent study [35], which showed that regional myocardial perfusion defects were present during periods of apnea in patients with OSA without obstructive CAD but were not present during daytime scintigraphy. In addition, the presence of cardiac risk factors in OSA patients, including insulin resistance, hypertension, and hyperlipidemia adversely, impairs vascular function. Cardiac risk factors are associated with increased oxidative stress [36]. Recruitment and differentiation of endothelial progenitor cells is also impaired by cardiovascular risk factors, preventing endothelial cell repair [37]. Finally, cardiac risk factors have been associated with increased asymmetric dimethylarginine [38], an endogenous eNOS inhibitor produced by increased methylation of L arginine, which decreases synthesis of NO, increases oxidative stress and inhibits endothelial cell repair.

### Study Limitations

A limitation of the study is the relatively small sample size, which may decrease the power to detect abnormalities in all clinical and imaging parameters. However, with the small sample size, we were able to detect the presence of microvascular disease and endothelial-dependent function, suggesting that these abnormalities are the most significant and earliest manifestation of CVD in patients with OSA. Further study in a larger population is warranted to confirm these findings. Furthermore, we may not be applying the most sensitive measures. Future studies, for example, could measure myocardial performance index, a more sensitive measure of ventricular function. In addition, 3 months of nCPAP may be inadequate to detect a significant change in some cardiovascular outcomes such as left ventricular mass, diastolic dysfunction, and pulmonary HTN. However, it may not be ethical to withhold nCPAP for a longer duration in this population. Future studies with a longer therapeutic duration could be performed in non-sleepy patients to determine if nCPAP is effective to improve these parameters. Another limitation of the study is that the duration of OSA may affect the results of CVI studies. Although we recruit only patients who are newly diagnosed, the duration that they had undiagnosed OSA is unknown. The final limitation of the study is that we are using imaging measurements as surrogate measurements for outcome. Findings from this initial study, however, may be applied to design a large, prospective randomized trial of nCPAP vs. sham CPAP to determine if nCPAP improves cardiovascular outcomes.

## Conclusions

Our findings indicate that nCPAP therapy improves coronary microvascular and endothelial function in OSA patients. These findings suggest that nCPAP therapy may prevent the development and progression of subclinical atherosclerosis, and, thus, significantly reduce cardiovascular morbidity and mortality in OSA patients. Larger, well-designed, multi-center prospective studies are needed to evaluate the impact of nCPAP therapy on various cardiovascular outcomes in OSA patients.

## Competing interests

The authors declare that they have no competing interests.

## Authors' contributions

PKN drafted the manuscript. PKN, CKK and PCY participated in the design of the study. PKN and CKK performed the MRI studies. PCY and CK conceived of the study, and participated in its design and coordination. MVM reviewed the manuscript. All authors read and approved the final manuscript.
